# Epidemiological Trends and Multidrug‐Resistant Patterns in Central Line‐Associated Bloodstream Infections Among Bone Marrow Transplant Patients at Comprehensive Cancer Center in Jordan: Five‐Year Retrospective Study

**DOI:** 10.1002/iid3.70495

**Published:** 2026-08-17

**Authors:** Sawsan Mubarak, Joud Jarrah, Hadeel AlGhawrie, Omar Khresat

**Affiliations:** ^1^ Infection Control Program King Hussein Cancer Center Amman Jordan; ^2^ Department of Medicine King Hussein Cancer Center Amman Jordan

## Abstract

**Background and Objective:**

Central Line‐Associated Bloodstream Infections (CLABSIs) considered a significant challenge in healthcare settings, necessitating vigilant monitoring and intervention strategies especially among patients undergoing Hematopoietic Stem Cell Transplantation (HSCT). This study aims to conduct comprehensive analysis of CLABSI cases among HSCT patients; trends in incidence, device associations, causative organisms, and multidrug‐resistant patterns.

**Methodology:**

A retrospective analysis was conducted on HSCT patients who developed CLABSI at the King Hussein Cancer Center between January 2019 and December 2023. Data collected included demographic data (age and sex), Diagnosis, type of vascular access device (VAD), Organism, bacterial resistance pattern, and last follow‐up status and date were collected for patients. Overall survival analysis was performed using the Kaplan–Meier method, with results reported at 30, 60, and 100 days post‐infection.

**Results:**

Over the 5‐year period, 169 HSCT patients had CLABSI were included, the majority were male (65.7%), with 73% adults and 27% pediatric patients, and a mean age of 33.6 years. The most common diagnoses were lymphoma (25.4%) and leukemias (24.9%). Gram‐negative bacteria were the predominant cause of infection, accounting for 85% of cases, followed by Gram‐positive bacteria (13.0%) and fungi (2.4%). *Escherichia coli* was the most frequent causative organism (46%), followed by *Klebsiella pneumoniae* (8%) and *Pseudomonas aeruginosa* (5%). Multidrug‐resistant pathogens, particularly Extended‐Spectrum Beta‐Lactamase (ESBL) producing strains and Carbapenem‐resistant *Enterobacteriaceae* (CRE), were prevalent. Hickman devices were associated with the highest number of CLABSI cases (63.9%). The overall survival rates were 83.4% at 30 days, 80.5% at 60 days, and 78.1% at 100 days post‐CLABSI.

**Conclusion:**

This study provides a comprehensive overview of CLABSI epidemiology among HSCT patients in Jordan, highlighting the high prevalence of Gram‐negative and multidrug‐resistant organisms. These findings underscore the critical need for continuous surveillance, targeted infection control measures, and effective antimicrobial stewardship to improve patient safety and outcomes in this high‐risk population.

## 1 Introduction

Hematopoietic Stem Cell Transplantation (HSCT) represents a life‐saving therapeutic intervention for various hematologic malignancies and bone marrow disorders. The King Hussein Cancer Center (KHCC) in Jordan is a high‐volume regional center, performing approximately 200 sophisticated blood and marrow transplants annually [1]. However, infectious complications remain a major source of morbidity and mortality in HSCT recipients, with bacterial infections posing particularly significant challenges for healthcare providers, hospital administrators, and patients [2, 3].

Among these infections, Bloodstream infections (BSIs) are noted as the most frequent infectious complications in this group, with incidence rates reaching 5%–10% in autologous‐HSCT and 20%–50% in allogeneic‐HSCT recipients, particularly before engraftment. For pediatric HSCT recipients, preventing Central Line‐Associated Bloodstream Infections (CLABSIs) is recognized as crucial for improving 1‐year survival rates [4].

Central Line‐Associated Bloodstream Infections (CLABSIs) are among the most serious device‐associated healthcare‐acquired infections. According to the Centers for Disease Control and Prevention (CDC), CLABSIs are defined as laboratory‐confirmed primary bloodstream infections in patients who had a central venous catheter in place for at least 48 h, where the infection is not related to another site. Central venous catheters are essential for HSCT patients, providing vascular access for chemotherapy, blood products, parenteral nutrition, and frequent blood sampling over extended periods. However, their prolonged presence can significantly increase the risk of serious infections [5, 6].

The clinical and economic burden of CLABSIs is substantial. These infections result in thousands of deaths annually and billions of dollars in healthcare costs, with individual infections costing up to $56,000 and prolonging hospital stays by 7.5–25 days. International surveillance data from the International Nosocomial Infection Control Consortium (INICC) reported CLABSI rates of 4.1 per 1000 central line days with crude mortality rates of 38.4% [7, 8].

HSCT patients face particularly elevated CLABSI risk due to several factors: prolonged periods of severe immunosuppression and neutropenia, extended catheter indwell times, frequent catheter manipulations, and underlying malignancies that compromise immune function [9].

Historically, Gram‐positive organisms have been the leading cause of CLABSIs in oncology and HSCT patients. However, recent trends indicate a rise in Gram‐negative pathogens, particularly in developing countries and among immunocompromised populations, posing significant challenges due to increasing antimicrobial resistance. This shift complicates empiric therapy and outcomes, especially given the rising prevalence of extended‐spectrum beta‐lactamase (ESBL)‐producing and carbapenem‐resistant organisms, Key pathogens often belong to the “ESKAPE” group that includes *Enterococcus faecium*, *Staphylococcus aureus*, *Klebsiella pneumoniae*, *Acinetobacter baumannii*, *Pseudomonas aeruginosa*, and *Enterobacter* spp [10, 11, 12, 13, 14].

Globally, CLABSI rates vary widely but have improved with strict bundle care protocols. Mortality associated with CLABSIs ranges from 12% to 25%, with higher rates reported in infections caused by multidrug‐resistant organisms. Device types also influence infection risk; Hickman catheters and Peripherally Inserted Central Catheter (PICC) are prominently associated with varied CLABSI incidence rates in HSCT settings [15, 16, 17, 18].

Previous research in Jordan focusing on pediatric autologous HSCT already demonstrated high bacterial infection rates, with Gram‐negative bacteria predominating [19]. Limited data exist regarding CLABSI epidemiology specifically in HSCT populations from the Middle East region. However, specific, comprehensive, and contemporary data mapping CLABSI epidemiology, device‐specific risk, causative organisms, and antimicrobial resistance patterns within the combined adult and pediatric HSCT cohort at the King Hussein Cancer Center (KHCC) are needed. Understanding the precise local ecological profile of CLABSI pathogens and resistance is imperative for evidence‐based refinement of local infection control protocols, antimicrobial stewardship efforts, and ultimately, enhanced patient safety.

The objective of this retrospective study was to comprehensively analyze Central Line‐Associated Bloodstream Infection (CLABSI) cases spanning a 5‐year period (January 2019–December 2023) among Hematopoietic Stem Cell Transplantation (HSCT) patients at the King Hussein Cancer Center. This investigation specifically aimed to determine the trends in incidence and device associations, identify the causative organisms and their detailed multidrug‐resistant patterns, and assess the resulting short‐term survival outcomes and associated mortality predictors.

## 2 Methods

### 2.1 Study Design and Setting

A retrospective observational study was conducted at King Hussein Cancer Center, a comprehensive cancer center in Amman, Jordan. The study analyzed patients who underwent Hematopoietic Stem Cell Transplantation (HSCT) and subsequently developed a Central Line‐Associated Bloodstream Infection (CLABSI) during the period spanned 5 years, from January 2019 to December 2023.

### 2.2 Study Population and Selection Criteria

The study included all patients who underwent HSCT, had a central venous catheter in place, and subsequently developed a CLABSI meeting CDC criteria. No sampling technique was employed; all eligible patients during the study period were included.

### 2.3 Data Collection

Data were collected from daily microbiology department reports maintained by the infection control office and verified through medical record review. Variables collected included patient demographics (age and sex), underlying diagnosis, and ward/unit location, type of vascular access device (VAD), causative organism, antimicrobial susceptibility patterns, and survival status with follow‐up dates.

According to the CDC guideline, CLABSI rates were calculated per 1000 central line‐days by dividing the number of CLABSI cases by the number of central line‐days and multiplying by 1000 [5].

### 2.4 Ethical Committee Approval

This study was conducted in accordance with the ethical standards of the Institutional Review Board (IRB) at the King Hussein Cancer Center (KHCC) in Amman, Jordan. Ethical approval for this research was formally granted by the KHCC IRB under the reference number 23 KHCC 173.

### 2.5 Statistical Analysis

Data were processed using Excel and analyzed using SPSS. Descriptive statistics, including means, medians, standard deviations, frequencies, and percentages were calculated for study variables. One‐way analysis of variance (ANOVA) was used to examine associations between device types and survival outcomes. Pearson chi‐square or Fisher exact tests were used for categorical variables, and significance was set at *p* < 0.05

The survival analysis begins by assessing the patient status (Alive or Dead) and calculating the overall survival duration from the date of infection diagnosis to the last follow‐up or death date. The Kaplan–Meier method is employed to perform the overall survival analysis, including survival by selected categorical variables such as Diagnosis Classification and Bacterial Classification. Results are reported at specific time points: 30, 60, and 100 days.

## 3 Results

### 3.1 Patient Demographics and Clinical Characteristics

Over the 5‐year study period, a total of 169 HSCT patients with CLABSIs were included in the analysis. The mean age of the cohort was 33.6 years, with ages ranging from 9 months to 68 years. The majority were male (65.7%, *n* = 111). Adults comprised 73.4% (*n* = 124) of the cohort, while pediatric patients represented 26.6% (*n* = 45).

The most common underlying diagnoses were lymphoma (25.5%, *n* = 43), acute myeloid leukemia (21.9%, *n* = 37), and acute lymphoblastic leukemia (17.8%, *n* = 30). Other diagnoses included aplastic anemia (11.2%, *n* = 19), myelodysplastic syndrome (8.3%, *n* = 14), multiple myeloma (7.1%, *n* = 12), myelofibrosis (2.4%, *n* = 4), chronic myeloid leukemia (1.8%, *n* = 3), neuroblastoma (1.8%, *n* = 3), and malignant infantile osteoporosis (2.4%, *n* = 4), which is illustrated in Tables 1 and 2.

**TABLE 1:** Patient characteristics and underlying malignancies (*N* = 169).

Variable	Frequency (%)
Gender	
Female	58 (34.3%)
Male	111 (65.7%)
Status	
Alive	104 (61.5%)
Dead	65 (38.5%)
Diagnosis classification	
Acute lymphoblastic leukemia (ALL)	30 (17.8%)
Acute myeloid leukemia (AML)	37 (21.9%)
Aplastic anemia	19 (11.2%)
Chronic myeloid leukemia (CML)	3 (1.8%)
Lymphoma	42 (24.9%)
Myelodysplastic syndrome	14 (8.3%)
Myelofibrosis	4 (2.4%)
Multiple myeloma	12 (7.1%)
Neuroblastoma	4 (2.4%)
Lymphoma	1 (0.6%)
Malignant infantile osteoporosis	3 (1.8%)
Diagnosis reclassification	
Lymphoma	43 (25.4%)
Multiple myeloma	12 (7.1%)
Myeloid disorders	18 (10.7%)
Others	26 (15.4%)
Leukemia	70 (41.4%)

**TABLE 2:** Patient age (*N* = 169).

Variable	*N*	Mean	Median	Min	Max	Range	SD
Age	169	33.6	35.0	0.58	68.0	67.4	19.7

### 3.2 CLABSIs Rate

**FIGURE 1:**
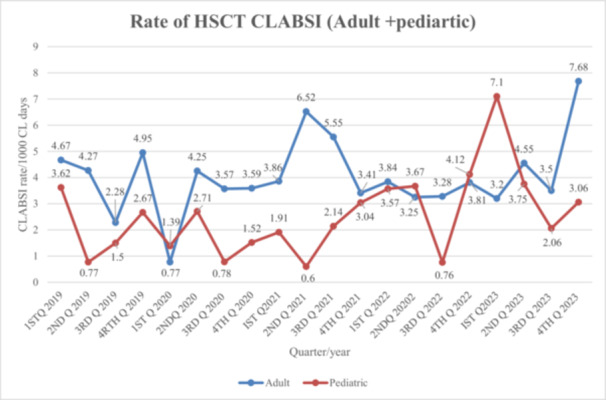
Quarterly rate of CLABSI among HSCT patients (2018–2023).

CLABSI rate was calculated quarterly as the previous formula. Figure 1 presents the quarterly rates of CLABSI among HSCT patients at King Hussein Cancer Center from 2018 to 2023. Rates are expressed per 1000 central line‐days, highlighting the fluctuations over time. Noticeable peaks are observed in Q1 2019 and Q4 2023, suggesting potential areas for targeted infection control interventions.

### 3.3 Device‐Specific Analysis

**FIGURE 2:**
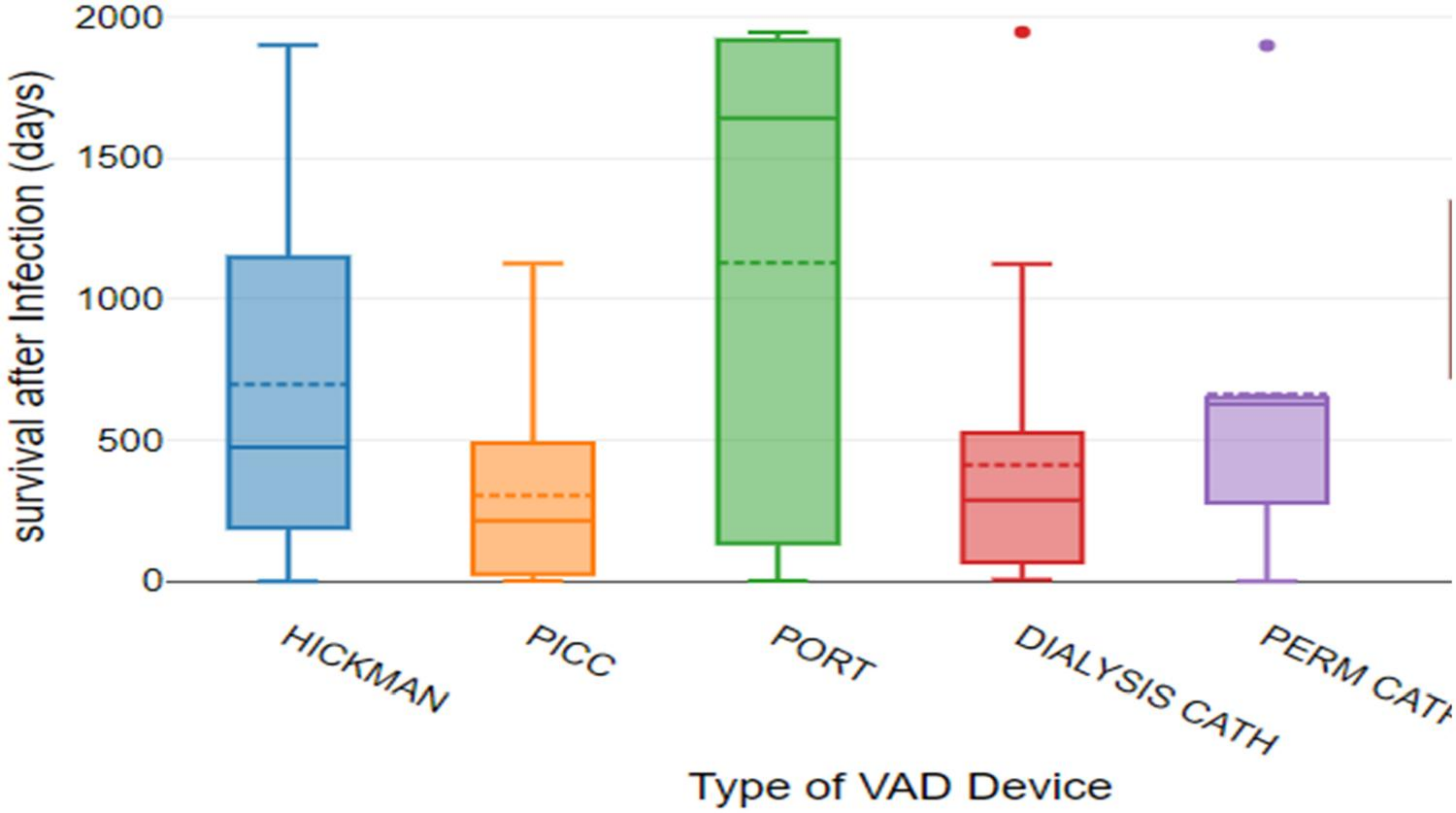
Survival after infection (days) by type of VAD device.

Device‐specific analysis revealed that Hickman catheters were the most common central line found in CLABSI cases (63.9%, *n* = 108), Peripherally Inserted Central Catheters (PICC) were the second most common device implicated (16.7%, *n* = 28). Other devices included port catheters (2.9%, *n* = 5), and central venous pressure (CVP) catheters (1.1%, *n* = 2), as showed in Table 3. A one‐factor analysis of variance has shown that there is a significant difference between the type of VAD device and the survival after infection (days) (*p* = 0.004, Figure 2).

**TABLE 3:** Type of VAD device (*N* = 169).

Type of VAD device	Frequency (%)
CVP	2 (1.2%)
Dialysis cath	20 (11.8%)
Hickman	108 (63.9%)
Perm cath	6 (3.6%)
PICC	28 (16.6%)
Port	5 (3.0%)

### 3.4 Microbiological Findings and Antimicrobial Resistance

Gram‐negative bacteria were the leading cause of CLABSIs, accounting for 85% of cases, while Gram‐positive bacteria caused 13.0% and fungi 2.4% of infections. *Escherichia coli* was the predominant pathogen, accounting for 45.6% of all cases. Among *E. coli* isolates, 45.4% were ESBL‐producing and 15.5% were carbapenem‐resistant *Enterobacteriaceae* (CRE). *Klebsiella pneumoniae* was the second most common organism, responsible for 17.8% of infections. Of these isolates, 50% were ESBL‐positive and 26.6% were classified as CRE. *Pseudomonas aeruginosa* represented 4.7% of isolates, with 12.5% being carbapenem‐resistant *Pseudomonas aeruginosa* (CRPA). *Acinetobacter baumannii* accounted for 2.4% of infections, all of which were multidrug‐resistant. Similarly, *Staphylococcus aureus* was isolated in 2.4% of cases, all identified as methicillin‐resistant *S. aureus* (MRSA). Overall antimicrobial resistance patterns showed that 29.0% of isolates were ESBL‐producing, 11.2% were CRE, 3.0% were multidrug‐resistant, 2.4% were MRSA, and 0.6% were CRPA, Table 4 illustrates the organis classification aligned with the most common organisms that are multidrug‐resistant (MDR).

**TABLE 4:** Organism classification.

Organism classification	Frequency (%)
Gram‐negative	143 (84.6%)
Gram‐positive	22 (13.0%)
Fungi	4 (2.4%)
*Escherichia coli*ESBL 35 (45.4%) CRE 12 (15.5%)	77 (45.6%)
*Klebsiella pneumoniae*ESBL 15 (50%) CRE 8 (26.6%)	30 (17.8%)
*Pseudomonas aeruginosa*CRPA 1 (12.5%)	8 (4.7%)
*Acinetobacter baumanni*MDR 4 (100%)	4 (2.4%)
*Staphylococcus aureus*MRSA 4 (100%)	4 (2.4%)

### 3.5 Clinical Outcomes and Survival Analysis

**FIGURE 3:**
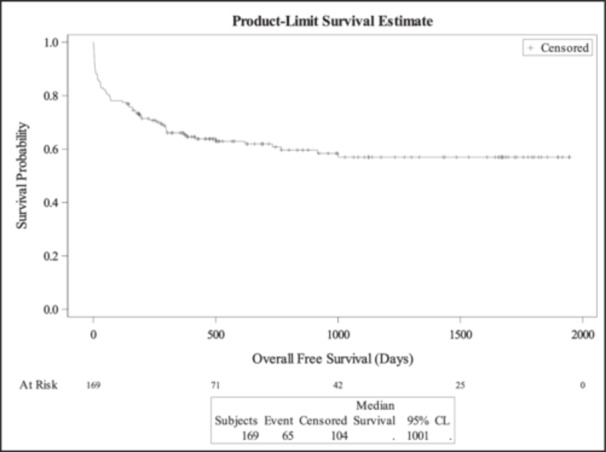
Overall survival rate (*N* = 169).

At the last follow‐up, 61.5% of patients were alive. Early mortality was significant, the overall 30‐day mortality rate for the entire cohort following CLABSI diagnosis was 17%. Kaplan–Meier survival analysis (Figure 3) revealed overall survival rates of 83.4% (95% CI: 77.5%–88.6%) at 30 days, survival slightly decreased to 80.5% (95% CI: 74.2%–86.1%) at 60 days, and 78.1% (95% CI: 71.6%–84.0%) at 100 days.

**FIGURE 4:**
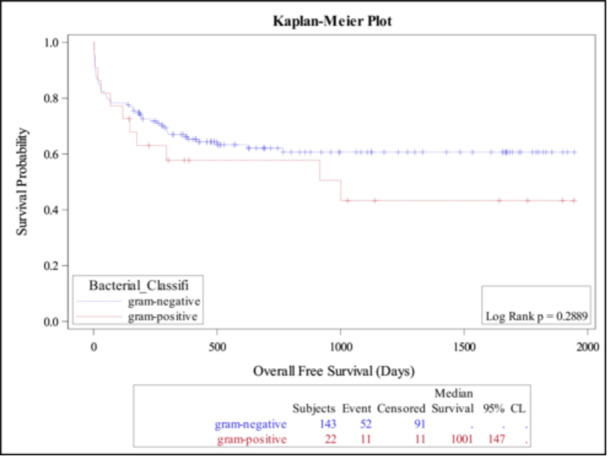
Overall survival by bacterial classification (*N* = 165).

When stratified by bacterial classification, Gram‐negative infections, Figure 4 showed survival rates of 83.2% at 30 days, 79.7% at 60 days, and 78.3% at 100 days. Gram‐positive infections demonstrated survival rates of 81.8% at both 30 and 60 days, and 77.3% at 100 days. Statistical analysis revealed a significant association between organism type and 30‐day mortality (*p* = 0.019), with *E. coli* associated with the highest mortality rates.

## 4 Discussion

This study provides a comprehensive overview of the epidemiology of CLABSIs among HSCT patients at a major cancer center in Jordan. The microbiological profile observed in this HSCT cohort marks a critical epidemiological finding for the region and the specific patient population. Evidence indicates a marked shift in the microbiological landscape of CLABSIs among HSCT recipients, with a predominance of Gram‐negative bacteria underpinned by rising antimicrobial resistance and a high prevalence of multidrug resistance. These trends complicate infection management and have direct impacts on patient survival.

The high incidence of Gram‐negative bacteria (85%) in our study is consistent with findings from other developing countries, where Gram‐negative bacteria are often the predominant cause of healthcare‐associated infections [20]. This contrasts with some reports from developed countries where Gram‐positive bacteria have historically been more common [21]. The most frequent pathogens identified in our study, *E. coli* and *K. pneumoniae*, are common causes of CLABSIs, but the high rates of resistance, particularly ESBL and CRE production, are alarming. These findings are in line with global trends of increasing antimicrobial resistance, which poses a significant challenge to the effective treatment of these infections [22]. This pattern validates the clinical necessity for local infection control protocols to focus intensely on minimizing the risk factors associated with gut translocation, such as dietary management and gastrointestinal tract integrity, and informs the initial selection of empirical antimicrobial therapy, demanding robust coverage against resistant Gram‐negative pathogens from the outset.

For the top two pathogens, ESBL rates approached 50%, and CRE rates reached 26.6% for *K. pneumoniae*. These findings confirm that MDRs are not merely occasional occurrences but are central to the infectious disease ecology within this transplant unit, demanding specialized antimicrobial agents. However, the high mortality associated with *E. coli* infection suggests that resistance within the highly prevalent Gram‐negative bacteria population is indeed the major driver of poor clinical outcomes. This underscores that effective treatment hinges critically on rapid identification and appropriate, targeted therapy against these prevalent ESBL/CRE strains [15, 18].

Notable finding that the Hickman device was associated with the highest frequency of CLABSI cases (63.9%), which is predictable given its frequent and prolonged use in HSCT patients requiring long‐term venous access. The lower rates of CLABSI associated with PICC lines in some studies were not observed here, suggesting that local factors and practices may play a significant role in device‐specific infection risks [23, 24]. The association between catheter type and infection rate emphasizes the need for stringent catheter care protocols tailored to device characteristics to minimize infection risk. Furthermore, the significant difference identified between the type of VAD device and survival after infection (*p* = 0.004) requires careful clinical consideration, this statistical association suggests either that the catheter type itself mechanically influences the ability to clear the infection or, more likely, that the choice of VAD serves as a proxy for the underlying severity of the patient's condition and treatment plan.

The overall survival rates in our cohort are comparable to those reported in other studies of CLABSIs in HSCT patients [25]. However, the 17% mortality rate within the first 30 days of infection underscores the severity of these infections and the need for prompt and effective management. The lack of a significant difference in survival between Gram‐positive and Gram‐negative infections in our study is interesting and warrants further investigation.

Regardless of the underlying causality, this finding emphasizes the necessity of strengthening device‐specific infection prevention bundles and potentially incorporating patient education and adherence contracts, which have been shown to reduce CLABSI rates elsewhere.

## 5 Study Limitations

This study is subject to several limitations inherent to its retrospective, single‐center design, which may limit the generalizability of findings, regional differences in antimicrobial resistance, and heterogeneity of clinical settings. The sample size was relatively small, which may have limited the statistical power to detect some associations.

## 6 Future Research Direction

Future research should alteration toward prospective, multicenter designs to enhance the generalizability of the results across diverse clinical settings and geographic regions. With Hickman and PICC lines frequently implicated in CLABSI cases, future research must evaluate the clinical impact of optimized, device‐specific catheter management protocols. Future studies should also evaluate the efficacy of tailored infection prevention bundles, optimizing the empirical antibiotic management and evaluating the effectiveness of continuous, rigorous surveillance systems.

## 7 Conclusion

This 5‐year epidemiological study from the King Hussein Cancer Center provides valuable insight into the CLABSI challenges facing HSCT recipients in Jordan. CLABSIs in HSCT patients are increasingly dominated by multidrug‐resistant Gram‐negative bacteria, particularly ESBL‐producing *E. coli* and *Klebsiella pneumoniae*. The frequent involvement of Hickman and PICC lines points to the need for optimized catheter management protocols. Given the substantial morbidity and mortality burden, strategic adjustments must focus on continuous, rigorous surveillance, targeted infection control bundles addressing the risks associated with the Hickman catheter, and proactive antimicrobial stewardship to optimize the empirical management of multidrug‐resistant Gram‐negative *Enterobacteriaceae*. These findings can help guide the development of targeted strategies to reduce the incidence of CLABSIs and improve outcomes for this vulnerable patient population.

## Author Contributions

**Joud Jarrah:** conceptualization, visualization, validation, writing – review and editing, project administration, supervision. **Hadeel AlGhawrie:** writing – original draft, methodology, writing – review and editing, formal analysis, data curation. **Omar Khresat:** methodology, validation, visualization, formal analysis, resources.

## Conflicts of Interest

The authors declare no conflicts of interest.

## Data Availability

The data that support the findings of this study are available from the corresponding author upon reasonable request.
